# Rheumatological Adverse Events Following Immunotherapy for Cancer

**DOI:** 10.3390/medicina58010094

**Published:** 2022-01-08

**Authors:** Ioana Cretu, Bogdan Cretu, Catalin Cirstoiu, Adrian Cursaru, Mihaela Milicescu, Mihai Bojinca, Ruxandra Ionescu

**Affiliations:** 1 Department Internal Medicine & Rheumatology, Carol Davila University of Medicine & Pharmacy, Dr Ion Cantacuzino Hospital, 917151 Bucharest, Romania; ghinia.ioana@gmail.com (I.C.); mihaela.milicescu@umfcd.ro (M.M.); mihai.bojinca@umfcd.ro (M.B.); 2 Department Orthopaedics & Traumatology, Carol Davila University of Medicine & Pharmacy, University Emergency Hospital, 050098 Bucharest, Romania; cirstoiu_catalin@yahoo.com (C.C.); adrian.cursaru@umfcd.ro (A.C.); 3 Department Internal Medicine & Rheumatology, Carol Davila University of Medicine & Pharmacy, Sf Maria Clinical Hospital, 011172 Bucharest, Romania; ruxandraionescu1@gmail.com

**Keywords:** immunotherapy, immune checkpoint inhibitors, immune-related adverse events, rheumatological adverse events

## Abstract

*Background and Objectives*: The occurrence of rheumatological side effects in a patient after receiving immunotherapy for cancer is becoming increasingly common. Oncologists often fail to diagnose and refer affected patients to rheumatologists. This paper presents the various rheumatological adverse events that occur after immunotherapy in patients as well as their treatment and evolution. *Materials and Methods*: A total of 36 patients were monitored between November 2018 and March 2020. The oncologist monitoring the immunotherapy-treated patients identified the occurrence of musculoskeletal side effects. The grading of toxicities was performed by both the oncologist and the rheumatologist using common terminology criteria for adverse events (CTCAE). Rheumatological treatment was administered, and for some patients, immunotherapy was discontinued. *Results*: The clinical presentations of the patients varied. Mild side effects (grade 1–2) were reported in a higher proportion than severe side effects (grade 3–5). Therefore, thirty-one patients had mild-to-moderate side effects, and five patients had severe side effects. Adverse reactions occurred, on average, 10 weeks after the initiation of immunotherapy; this indicated that the severity of the toxicity was dose dependent. Patients were treated with NSAIDs or prednisone, depending on the severity of the side effects, and for patients with severe manifestations, immunotherapy was discontinued. The remission of rheumatic manifestations varied depending on the grade of the manifestations. *Conclusions*: The clinical, biological, and ultrasound presentations of the patients with adverse events followed by cancer treatments differed from classic rheumatological manifestations. Thorough examinations of these patients by both oncologists and rheumatologists are needed in order to correctly diagnose and treat rheumatological adverse events. Multiple studies that include a larger number of participants are needed in order to better understand the pathogenesis and clinical evolution of these patients under different treatment conditions.

## 1. Introduction

Oncological therapy that uses immune pathways to obtain an antitumor response has been the subject of much research and has been frequently used to treat neoplasms in recent years [1]. The basic concept underlying immunotherapy is to activate T lymphocytes that will attack tumor cells. Subsequently, molecules that control T-cell activation were discovered, namely cytotoxic T-lymphocyte-associated antigen-4 (CTLA-4) and programmed death-1 (PD-1)/programmed death ligand-1 (PD-L1). The discovery of these molecules has altered the treatment of certain cancers based on the development of compounds that target these proteins. For example, ipilimumab and tremelimumab target CTLA-4, nivolumab and pembrolizumab target PD-1, and durvalumab and atezolizumab target PD-L1 [2].

Immunotherapy is one of the most important developments in medicine in recent years, but with the advent of these treatments, adverse reactions such as immune-related adverse events (irAE) have been reported [3]. The greater the number of patients treated with immunotherapy, the greater the number of side effects, including rheumatic side effects [2].

Immunotherapy works on the inhibitory pathways of the immune system in different stages. CTLA-4 is an early signal inhibitor during the primary phase of T-lymphocyte activation [4]. PD-1 is a receptor present on the surface of several immune cells such as T lymphocytes, B lymphocytes, and NK lymphocytes, and it binds to either ligand 1 or 2 (PD-L1, PD-L2), which are exposed to tumor cells and cause apoptosis [5]. At this stage, either anti-PD-1 or anti-PD-2 acts by destroying the immunotolerance, which leads to the destruction of tumor cells by T lymphocytes [6].

Given the different mechanisms of action of anti-CTLA4 therapy versus anti-PD-1/L1, we also encounter different immune toxicities. This was first demonstrated in murine models. Mice receiving anti-CTLA-4 therapy had fatal lymphoproliferation, while those receiving anti-PD-1 therapy had fewer toxicities, such as arthritis and lupus-like glomerulonephritis. However, in humans, anti-CTLA-4 therapy is more toxic than PD-1/L1 [7].

The mechanism of toxicity has been investigated. However, the clinical and paraclinical data reported thus far have suggested that immunological adverse reactions occur due to the disruption of homeostasis. This balance is maintained by T lymphocytes, but other cells of the immune system are also involved. Immunotherapy, however, causes aberrant T-cell activation both against tumor cells and against normal tissues. PD-1 is present on the surface of B cells and plays an important role in the humoral immune response. Hence, anti-PD-1/L1 therapy can modulate antibody production by interfering with PD-1, which may induce cytokine-mediated toxicity. For example, ipilimumab-induced colitis leads to an increased production of IL-17, a proinflammatory cytokine, by helper T cells [8].

Almost all organs can be affected by immunotherapy, with side effects ranging from mild to life threatening. The skin, colon, endocrine organs, liver, and lungs are the most commonly affected [9]. Some side effects are rare but very severe, such as myocardial infarction or neurological disorders. In general, side effects appear within the first three weeks and up to a few months after starting treatment, but there have been cases in which they appeared after one year [8,9].

Rheumatological/musculoskeletal side effects are becoming more common, and rheumatological toxicities have not always been thoroughly described by oncologists. The initial reports of adverse reactions vaguely described musculoskeletal adverse reactions or reported only severe adverse reactions [10]. While oncological reports showed the incidence of rheumatic adverse reactions to be less than 1%, clinical data indicate a higher incidence, at approximately 5% [11]. In addition, patients with autoimmune disorders were excluded from the studies, so there was very few data for these patients [12].

Rheumatological side effects are a challenge, both regarding their recognition and treatment. Questionnaires completed by rheumatologists in France in 2018 revealed that 70% of them were not familiar with the toxicities induced by immunotherapy, and 90% indicated they did not feel prepared to treat these diseases [13].

Rheumatological toxicities differ from the toxicities of other organs. They persist for longer after the cessation of oncological treatment and are widely affected by arthralgia, inflammatory arthritis, rheumatic polymyalgia, myalgia/myositis, and Sjogren’s syndrome, as well as lupus-like vasculitis and sarcoidosis. These side effects have been reported in patients who have no history of autoimmune disease. However, there have also been reports of patients with autoimmune diseases who have experienced outbreaks after immunotherapy [14]. Given that these manifestations can be treated, the presence of an autoimmune disease is not contraindicated for these oncological treatments [15].

After a patient has been diagnosed with rheumatological toxicity induced by immunotherapy, they should be prescribed the optimal treatment in considering the patient’s quality of life and the possibility of continued oncological treatment [16].

Rheumatological side effects after immunotherapy represent a new clinical challenge, especially given that a large proportion of patients do not meet the diagnostic criteria for known rheumatic diseases, though some patients do present with classic rheumatic diseases. Rheumatologists should be familiar with these manifestations in order to treat them correctly [17]. In addition, paraneoplastic manifestations and metastases can present with musculoskeletal pain. This should be considered, especially in patients who have not responded to treatment or in those whose manifestations have been accentuated during treatment. In these cases, the rheumatologist must carefully analyze data for the medical history laboratory testing and imaging of the patient [18].

This study aims to analyze patients treated with immunotherapy where rheumatological adverse events occurred in addition to examining the treatment and evolution of these events over a period of time. We present various forms of rheumatic events and important data to guide future diagnosis and treatment.

## 2. Materials and Methods

A total of 36 patients were monitored between November 2018 and March 2020 in a retrospective cohort study. The oncologist monitoring the patients, who had been treated with immunotherapy, identified the occurrence of musculoskeletal side effects. Only oncological patients that presented with rheumatological manifestations after initiating treatment with monoclonal antibodies were included in the study. None of the patients had been diagnosed with an autoimmune disease prior to beginning their cancer treatments. All patients were treated for neoplastic disease with either anti-PD-1 (nivolumab and pembrolizumab) or anti-PD-L1 (atezolizumab). There were 348 patients receiving immunotherapy and the incidence of rheumatic adverse events was 10.34%. Retrospective case series reported rheumatological adverse events such as arthralgia, arthritis, seronegative arthritis, and myositis, with an incidence of 3.5–13% [19].

The grading of toxicities was performed by both the oncologist and the rheumatologist. In oncology, adverse immunological reactions are described using common terminology criteria for adverse events (CTCA), which assesses severity on a scale of 1 to 5 (1—mild, 2—moderate, 3—severe, 4—life-threatening, 5—toxicity with a high mortality rate). All patients were over 18 years old. They received treatment with ipilimumab, pembrolizumab, and atezolizumab for metastatic melanoma, small-cell lung cancer, urothelial carcinoma, and renal cell carcinoma. The rheumatologist clinically evaluated the patients in terms of joint pain, morning stiffness, joint swelling, and muscle pain. A musculoskeletal ultrasound was performed on joints that were reported to be in pain. The combined EULAR–OMERACT score was applied to grade rheumatoid arthritis synovitis. Following the ultrasound examination, numerous changes were highlighted. Hand and knee radiographs were performed in patients with pain at this level. Radiography was needed for a better description of the joint and to explore diagnostic alternatives. After clinical and imaging evaluations, varied manifestations were identified: inflammatory arthritis, arthralgia, myalgia, rheumatoid arthritis, and Sjogren’s syndrome.

The following information was entered into the database: age of patients, sex, type of malignancy, type of treatment, duration of treatment until adverse reactions occurred, serology (CRP, ESR, FR), type of toxicity, gradation of adverse reactions, response to treatment, and if it was necessary to discontinue cancer treatment. The response to treatment was assessed as positive in patients who no longer had musculoskeletal manifestations or if they had mild manifestations that no longer required treatment. The response was assessed as negative in patients who had continuous joint pain after the end of rheumatological treatment. The occurrence of side effects in other organs was also considered, given that dermatological, endocrine, and gastrointestinal damages were more common than musculoskeletal manifestations.

## 3. Results

A total of 36 patients, 23 males and 13 females, were evaluated. Within this limited sample, rheumatic side effects were more common in males. The average age of patients was 61 years old. The youngest was 40 years old and the oldest patient was 79 years old. The people included in the study did not have significant pathologies other then neoplasm. Most were receiving treatments for hypertension, dyslipidemia and hypothyroidism.

Nineteen patients received nivolumab, fourteen patients received pembrolizumab, and three patients received atezolizumab. Twenty-one patients were diagnosed with metastatic melanoma, ten patients with non-small-cell lung cancer, three patients with urothelial carcinoma, and two patients with renal cell carcinoma (Table 1). Most of the patients with metastatic melanoma had radiocubitocarpian, metacarpofalangia and knee arthralgia. Two patients presented with subacromial–subdeltoidian bursitis, and one of them had knee arthritis and myalgia. Another patient presented with radiocubitocarpian and metacarpofalangian arthritis. One patient was diagnosed with Sjogren syndrome. Among the patients with non-small-cell lung cancer, one was diagnosed with rheumatoid arthritis, another one had xerostomia and xerophthalmia and one patient had radiocubitocarpian and metacarpofalangia arthritis. The rest of the patients had arthragias of knee, shoulders, radiocubitocarpian and metacarpofalangian joints.

The time from the start of treatment to the onset of side effects was, on average, 10 weeks (a range of 2–20 weeks). Of the examined patients, three had other side effects before the onset of rheumatology. They presented with dermatological manifestations (i.e., erythema, atopic dermatitis, and pruritus) that appeared approximately two weeks after initiating antibody treatment.

### 3.1. Musculoskeletal Toxicity after Immunotherapy

Mild side effects (grades 1–2) were reported in a higher proportion than severe side effects (grade 3–5). In total, thirty-one patients had mild-to-moderate side effects and five patients had severe side effects. It was observed that there was a higher incidence of side effects after nivolumab. The most severe manifestations were also in the patients treated with nivolumab.

Most patients had arthritic changes in the joints, both according to clinical and imaging evaluations, but did not experience joint pain before starting cancer treatment.

The clinical presentations of the patients were varied. Joint pain occurred in both large and small joints. The large joints that showed pain were the shoulders, elbows, knees, and tibiotarsal joints. The small joints that showed pain were the radio-cubito-carpal, metacarpophalangeal, proximal interphalangeal, and distal interphalangeal.

Shoulder pain occurred in four of the patients. Two of them presented with bilateral subacromial–subdeltoid bursitis and bilateral bicipital tendonitis. Only one patient had elbow pain. Knee pain occurred in fifteen patients, two of whom had moderate synovitis. Only one patient had moderate synovitis in the tibiotalar joint.

**Figure 1:**
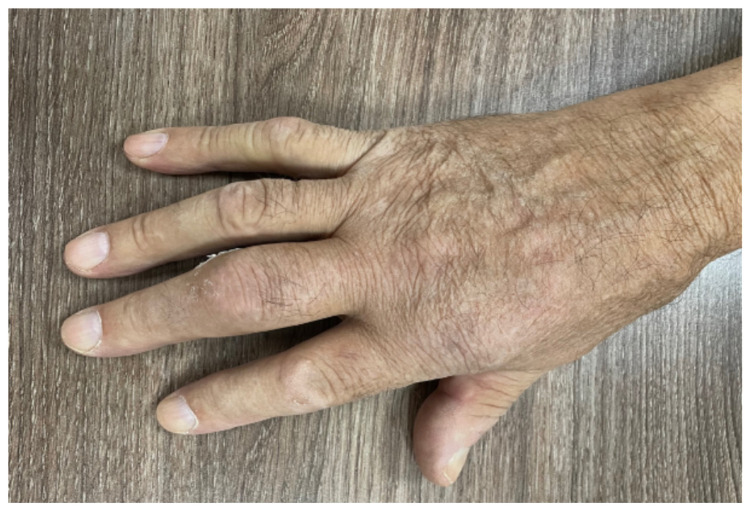
Patient with arthritis and tenosynovitis.

Ten patients presented with pain in the small joints of the hands. Following musculoskeletal ultrasound, it was observed that five of the patients had moderate synovitis in the radio-cubito-carpal and metacarpophalangeal joints, with a grade I–II Doppler signal. Of the five patients with inflammatory arthritis, one was diagnosed with seronegative rheumatoid arthritis. In addition, two other patients presented with arthritis in the RCC as well as tenosynovitis in the tendons of the hand extensors (Figure 1).

Five patients had severe myalgia without altered muscle enzymes. Myalgia occurred in the upper and lower limbs, and one of the patients also had extrapyramidal syndrome associated with myalgia. In these cases, it was necessary to exclude polymyositis/dermatopolymyositis, which may be a paraneoplastic manifestation.

The rheumatoid factor was analyzed in all patients; it was negative in all cases. Acute phase reactants, ESR, and CRP values were analyzed; however, they often have high values in oncological disease and, therefore, may not be a good indicator of rheumatic activity. However, the greatest inflammatory syndrome was found in the most severe cases. ANA was also collected from all patients. Only one patient presented as ANA positive, and an extended ANA profile showed anti-Ro positive antibodies. This patient had xerophthalmia and xerostomia and was diagnosed with Sjogren’s syndrome.

Adverse reactions occurred, on average, 10 weeks after the initiation of immunotherapy. One patient presented with rheumatological manifestations 2 weeks after the first administration. At the latest, it appeared 20 weeks after the first administration, but before toxicity occurred, a double dose of nivolumab was administered. This indicates that the severity of the toxicity was dose dependent.

### 3.2. Treatment of Rheumatic Side Effects

Patients were treated with NSAIDs or prednisone, depending on the severity of the side effects. Attempts have been made to treat patients with the lowest possible dose of corticosteroids in order to maintain immunotherapy for as long as possible. If treatment was discontinued, the presence of adverse reactions in other organs was also considered. One patient was diagnosed with seronegative rheumatoid arthritis after immunotherapy and was treated with dexamethasone for 5 days with symptom relief and then received 15 mg/day of prednisone and sulfasalazine. Another patient with inflammatory arthritis in the small joints of the hands required sulfasalazine and prednisone. This patient also presented with hyperuricemia, so treatment with colchicine, and later with milurite, was initiated.

Patients with mild manifestations received NSAID treatment with symptom relief after approximately two weeks. Patients with moderate manifestations were treated with 10 mg/day prednisone, with a dose reduction after 2 weeks of treatment. No patients showed increased symptoms after lowering corticosteroid doses.

In patients with severe manifestations, immunotherapy was discontinued, and high doses of prednisone were introduced. The remission of rheumatic manifestations appeared after about 4 weeks. Of the patients with severe manifestations, only four resumed treatment with nivolumab/pembrolizumab. One of the patients with severe manifestations did not receive treatment with nivolumab due to the tumor progression under treatment but also due to the persistence of joint pain. Another two patients discontinued nivolumab for an indefinite period due to joint pain and myalgia that did not completely resolve after the discontinuation of cancer treatment and the initiation of prednisone/sulfasalazine therapy (Table 2).

Comparing the treatment of inflammatory arthritis in patients who did not have neoplasms and those who had side effects after immunotherapy, we found that the latter received treatment with corticosteroids and not with disease-modifying antirheumatic drugs (DMARDs). This is because these patients responded well to steroid treatment but also because we did not have enough experience in using DMARDs in these situations.

## 4. Discussions

Rheumatological toxicities should be recognized by the oncologist and treated together with the rheumatologist [20]. At each cancer evaluation, patients receiving immunotherapy should be asked if they have joint or muscle pain, morning stiffness, and xerophthalmia/xerostomia, as well as diffuse pain that could suggest fibromyalgia [19].

In this study, we identified 36 patients who had rheumatological side effects. Nineteen of them were treated with anti-PD1 antibodies and seventeen with anti-PD-L1 antibodies. The average time of onset was 10 weeks, but in other studies, it was about 12 weeks [21]. Rheumatological side effects occurred later after the initiation of immunotherapy compared to side effects in other organs (for example, dermatological side effects that started in the first weeks after starting treatment).

There were two patients who presented with xerostomia and xerophthalmia, and one of them fulfilled the criteria of ACR/EULAR group 2017 for true Sjogren syndrome. The REISAMIC registry reported 4 among 908 patients with Sjogren Syndrome who had received anti PD1/PD-L1 therapies. [22].

In terms of treatment, patients responded well to treatment with NSAIDs and prednisone. From existing data, symptoms should resolve within 6–12 weeks of corticosteroid treatment. In severe cases, biological treatment can be given, but these treatments could not be administered to patients in Romania at this particular time. Treatments with DMARDs should be considered in patients who do not respond to corticosteroid therapy. The decision to discontinue cancer treatment, whether for a certain period of time or permanently, should be made very carefully and discussed with both the patient and the oncologist.

There are many reasons why patients with musculoskeletal manifestations that begin after immunotherapy are not referred to a rheumatologist. First of all, oncologists do not have a questionnaire for the correct identification of these problems and, therefore, they are often overlooked. Another reason would be that the symptoms are alleviated by corticosteroid treatment that the patient may receive for other side effects (especially if they occurred earlier). Another problem is that the patients do not specify rheumatic pain due to the presence of many symptoms related to neoplasia.

In the case of musculoskeletal pain, metastases and paraneoplastic manifestations should be considered to allow a differential diagnosis of rheumatic side effects after immunotherapy to be made.

At this time, the actual incidence of rheumatic adverse reactions is unknown, as is whether there are there any risk factors that predispose a patient to their occurrence [17,18]. The clinical, biological, and imaging presentations of patients are varied, and more research is needed to correctly diagnose these patients.

Oncological treatments are constantly evolving and the latest studies show us that combining anti-angiogenetic therapy and immunotherapy seems to have the potential to tip the balance of the tumor microenvironment and improve treatment response [23].

## 5. Strengths and Limitations of the Study

A strength of this study is the fact that it describes a variety of rheumatic conditions that may occur after immunotherapy. Additionally, the treatment was adapted according to the clinical, biological and imagistic presentations of the patients. A limitation of the study is that a small number of patients were evaluated.

## 6. Conclusions

In this study, the numerous musculoskeletal side effects that occur after immunotherapy were highlighted. Patients with arthralgia, oligoarthritis/inflammatory polyarthritis, tenosynovitis, myalgia, rheumatoid arthritis, and Sjogren’s syndrome were identified. The clinical, biological, and ultrasound presentations of the patients with adverse events followed by cancer treatments differed from classic rheumatological manifestations.

Musculoskeletal side effects after immunotherapy were found to be more common than previously thought. We note that these pathologies did not meet the criteria for classification as rheumatic diseases, so we may be dealing with a new category of musculoskeletal disorders.

A thorough examination of these patients by both oncologists and rheumatologists is needed. A rheumatological evaluation of patients receiving immunotherapy with articular and muscular manifestations is needed for better characterizing and treating rheumatological adverse events. Currently, patients are only treated by a rheumatologist according to their self-reported symptoms and, in collaboration with the oncologist, the specialists then make a decision regarding the optimal therapy. A common guide is needed in order to diagnose and treat possible adverse events that are secondary to immunotherapy.

Multiple studies that include a larger number of participants are needed in order to better understand the pathogenesis and clinical evolution of these patients under different treatment conditions.

## Figures and Tables

**Table 1:** Rheumatological adverse events specific to cancer type.

	Metastatic Melanoma	Non-Small-Cell Lung Carcinoma	Urothelial Carcinoma	Renal Cell Carcinoma
Nivolumab	13	6	0	0
Pembrolizumab	7	4	3	0
Atezolizumab	1	0	0	2

**Table 2:** Rheumatological adverse events secondary to immunotherapy, treatment, and management.

Rheumatological Adverse Reactions	Treatment	Immunotherapy
Arthralgia	NSAID	Continued
Myalgia	NSAID, prednisone 5–10 mg/day	Continued
Inflammatory arthritis, tenosynovitis, bursitis	Prednisone 10–20 mg/day	Discontinued until symptom remission
	DMARDs	
Sjogren syndrome	Prednisone 5–10 mg/day	Continued
Seronegative rheumatoid arthritis	Prednisone 10–20 mg/day	Discontinued indefinitely
	DMARDs	

## Data Availability

Further data concerning the study can be obtained by contacting the corresponding author.
